# Gamma oscillations induced by 40-Hz visual-auditory stimulation for the treatment of acute-phase limb motor rehabilitation after stroke: study protocol for a prospective randomized controlled trial

**DOI:** 10.1186/s13063-024-08121-w

**Published:** 2024-04-26

**Authors:** Wang Fu, Xiaoming Yu, Minghui Lai, Yuanli Li, Yingting Yang, Yong Qin, Min Yu, Feng Wang, Cong Wang

**Affiliations:** 1 Department of Neurology, Seventh People’s Hospital of Shanghai University of Traditional Chinese Medicine, Datong Rd. 358, Shanghai, 200137 China; 2 Department of Rehabilitation, Seventh People’s Hospital of Shanghai University of Traditional Chinese Medicine, Shanghai, 200137 China; 3 Engineering Research Center of Traditional Chinese Medicine Intelligent Rehabilitation, Ministry of Education, Shanghai, 201203 China; 4 School of Rehabilitation Science, Shanghai University of Traditional Chinese Medicine, Shanghai, 201203 China; 5 Queensland Brain Institute, the University of Queensland, Brisbane, 4072 Australia

**Keywords:** Stroke, Upper extremity impairment, Gamma oscillations, 40-Hz visual-auditory stimulation, Randomized controlled trial

## Abstract

**Background:**

The incidence of hemiparetic limb dysfunction reaches 85% in stroke patients, emerging as a critical factor influencing their daily lives. However, the effectiveness of current rehabilitation treatments is considerably limited, particularly in patients with upper extremity impairment. This study aims to conduct a prospective clinical trial to validate the safety and effectiveness of gamma oscillations induced by 40-Hz visual-auditory stimulation in treating post-stroke upper limb dysfunction and to explore the relevant mechanisms.

**Methods:**

This trial is a prospective, randomized controlled, double-blind study. All enrolled patients were randomly assigned to two groups. The experimental group received intervention through 40-Hz visual-auditory stimulation, while the control group underwent intervention with randomly matched visual-auditory stimulation frequencies. The primary efficacy endpoint is the change in motor function. Secondary efficacy endpoints include motor-evoked potentials, cerebral hemodynamic changes, neural network connectivity, and alterations in synaptic-related genes. Safety evaluation included major adverse events, all-cause mortality, and photosensitive epilepsy. Assessments will be conducted at baseline, after a 14-day treatment period, and during subsequent follow-up visits (at 3 and 6 months) post-treatment. The differences between the two groups will be compared.

**Discussion:**

This study will evaluate the safety and efficacy of gamma oscillations induced by 40-Hz visual-auditory stimulation in treating patients with upper extremity dysfunction after an acute cerebral stroke. Concurrently, we will explore potential mechanisms, including changes in synaptic-related genes and neural network connectivity. This trial is expected to provide evidence for the effectiveness of this new technique in treating upper extremity dysfunction after a stroke and improving patients’ quality of life.

**Trial registration:**

The study protocol has been registered with the Chinese Clinical Trial Registry (ChiCTR) under registration number ChiCTR2300076579 on October 12, 2023.

## Administrative information

Note: the numbers in curly brackets in this protocol refer to SPIRIT checklist item numbers. The order of the items has been modified to group similar items (see http://www.equator-network.org/reporting-guidelines/spirit-2013-statement-defining-standard-protocol-items-for-clinical-trials/).

**Table:** 

Title {1}	Gamma oscillations induced by 40-Hz visual-auditory stimulation for the treatment of acute-phase limb motor rehabilitation after stroke: study protocol for a prospective randomized controlled trial
Trial registration {2a and 2b}.	• This protocol has received approval from the Ethics Committee of the Seventh People's Hospital of Shanghai (Approval Number: 2023-7th-HIRB-027) and will be conducted following the ethical standards outlined in the Helsinki Declaration. Additionally, the study protocol has been registered with the Chinese Clinical Trial Registry (ChiCTR) under registration number ChiCTR2300076579 on October 12, 2023.
Protocol version {3}	V1.0, Oct 12, 2023
Funding {4}	This work is supported by the National Natural Science Foundation of China (Grant No. 82202787), Frontier Innovative Talents of Traditional Chinese Medicine Program of Shanghai University of Traditional Chinese Medicine (Grant No. 009), Shanghai Municipal Science and Technology Commission (Grant No. 23Y11906400), Research Project of the Shanghai Municipal Health Commission (Grant No. 20234Y0241), Outstanding Leaders Training Program of Pudong Health Bureau of Shanghai (Grant No. PWR12020-03), Pudong New Area Science and Technology Development Fund for Livelihood Research Special Project (Grant No.PKJ2023-Y60). Discipline Construction of Pudong Health Bureau of Shanghai—Discipline group of cerebrovascular system diseases (Grant No. PWZxq2022-01).
Author details {5a}	Wang Fu^*1*^*†*, Xiaoming Yu^*2*^*†*, Minghui Lai^*2*^, Yuanli Li^*2,3,4*^, Yingting Yang^*1*^, Yong Qin^*1*^, Min Yu^*1*^, Feng Wang^1^*, Cong Wang^1,3,4,5^* *1 Department of Neurology, Seventh People’s Hospital of Shanghai University of Traditional Chinese Medicine, Shanghai, 200,137, China* *2 Department of Rehabilitation, Seventh People’s Hospital of Shanghai University of Traditional Chinese Medicine, Shanghai, 200,137, China* *3 Engineering Research Center of Traditional Chinese Medicine Intelligent Rehabilitation, Ministry of Education, Shanghai, 201,203, China* *4 School of Rehabilitation Science, Shanghai University of Traditional Chinese Medicine, Shanghai, 201,203, China* *5 Queensland Brain Institute, the University of Queensland, Brisbane, 4072, Australia*
Name and contact information for the trial sponsor {5b}	Department of Neurology, Seventh People’s Hospital of Shanghai University of Traditional Chinese Medicine, Datong Rd. 358, Shanghai 200,137, China. Phone: 86–21-58,611,047
Role of sponsor {5c}	The sponsor played no role in the study design, manuscript writing, data collection, management, analysis, and interpretation. The sponsor did not contribute to the decision to submit this report for publication and does not have ultimate authority over any of the authors’ activities.

## Introduction

### Background and rationale {6a}

Stroke remains the second-leading cause of death all over the world, characterized by a high incidence, high fatality rate, significant disability rate, and a high rate of recurrence. From 1990 to 2019, the absolute number of incident strokes globally increased by 70.0%, prevalent strokes increased by 85.0%, and deaths due to stroke increased by 43.0% [1, 2]. Stroke imposes a tremendous economic burden globally. It is reported that in high-income countries, household income loss due to stroke accounts for approximately one-third, while in upper-middle-income countries, it amounts to around half. In low-income countries, income losses attributed to stroke represent about 15% of the global total (largely due to their significantly lower income levels compared to high-income countries) [3]. Acute ischemic stroke, constituting approximately 80% of all strokes, presents a therapeutic challenge with suboptimal outcomes. Despite significant breakthroughs in vascular reperfusion techniques, including thrombolysis and thrombectomy, in the treatment of ischemic stroke [4, 5], the limited treatment window (benefiting less than 3% of patients) [6] and constraints of mechanical thrombectomy technology (with a reperfusion rate of 13 to 18%) [7] result in limited benefits and poor prognosis, particularly for patients with major vessel occlusion or severe conditions. Ongoing efforts in medical practice focus on refining thrombolysis and mechanical thrombectomy techniques, aiming to enhance patient outcomes [8, 9].

Due to the suboptimal efficacy of stroke treatment, the disability rate associated with stroke is high. Approximately 50 to 70% of patients with stroke endure varying degrees of functional impairments, encompassing muscle weakness, sensory deficits, cognitive impairments, etc., profoundly impacting their quality of lives [10, 11]. Among them, the incidence of hemiparetic limb dysfunction reaches 85%, emerging as a critical factor influencing the therapeutic aspects of patients’ daily lives [12]. Therefore, post-stroke rehabilitation is of paramount importance. As early as the AHA/ASA guidelines in 2016, it emphatically underscored the necessity for training and education of caregivers in providing stroke patients with a comprehensive rehabilitative treatment plan [13]_._ Plenty of rehabilitation approaches are currently available, including conventional rehabilitation, mirror therapy, and neuroregulatory techniques. However, the treatment effect is quietly limited. Recently, neuromodulation techniques have gained increasing attention. Among them, repetitive transcranial magnetic stimulation (rTMS) has been reported to be effective in treating post-stroke limb function disability [14]. Unfortunately, the rehabilitation outcomes for upper limb dysfunction in post-stroke patients are suboptimal, constituting a focal and challenging aspect of current rehabilitation efforts. Approximately 33 to 66% of people with upper extremity (UE) impairments partially recover after treated by rTMS at 6 months, but only 5–20% fully recover [15]. Possibly, the unclear selection of target areas for rTMS and the lack of clarity in the relationship between neural circuit activation and reconstruction have limited its effectiveness in treating post-stroke upper limb dysfunction. Therefore, there is an urgent need to identify a safer, more effective, economical, and convenient method to enhance the rehabilitation outcomes for upper limb dysfunction.

In recent years, the notion of “gamma frequency stimulation inducing gamma neural oscillations to enhance brain function recovery” has evolved into a groundbreaking discovery within the field of neuroscience. In the year 2002, the reduction in synchronicity of gamma wave oscillations in the brains of Alzheimer’s disease (AD) patients was first reported [16]. Furthermore, genetic modifications [17] and optogenetics [18] have also been confirmed to establish a relationship between induced gamma oscillations and behavioral effects in AD mouse models. Balbi et al. found that 40-Hz optogenetic stimulation-induced gamma neural oscillations, thereby promoting motor function recovery in post-stroke mice [19]. The same conclusion has been reached by our team, and the underlying mechanism has been verified to involve rescuing functional synaptic plasticity [20]. Owing to its reliance on genetic manipulation techniques, optogenetics had been circumscribed in its clinical translational applications. Tsai et al. initially affirmed that non-invasive method, specifically 40-Hz visual-auditory stimulation, could induce gamma oscillations in the visual cortex of AD mouse models, thereby diminishing the burden of amyloid-like proteins in the brain [21]. Subsequently, they further validated that this light flicker stimulation effectively decelerates neuronal degeneration, preserved synaptic functionality, mitigated inflammatory responses in microglial cells, and enhanced memory functions in AD mice. Furthermore, similar effects were observed in auditory stimulation and combined visual-auditory stimulation [22]. Hou et al. found that the utilization of 40-Hz gamma frequency light stimulation effectively reinstated slow gamma neural oscillations in the hippocampal region of mice post-stroke and confirmed the causative relationship between gamma oscillations and post-stroke cognitive impairments. Furthermore, they concluded that this stimulatory regulatory approach safeguarded ischemic neurons by enhancing synaptic plasticity and associated long-term potentiation [23].

Based on previous foundational research, gamma oscillations show promising potential in promoting brain function recovery. Among them, 40-Hz visual-auditory stimulation is safe, is well-tolerated, and holds the greatest clinical translational prospects. As early as 2002, the 40-Hz auditory stimulation was applied to healthy individuals, revealing an augmentation in localized cerebral blood flow and substantiating the safety of 40-Hz stimulation [24]. In recent years, numerous clinical investigations had reported on the amelioration of brain function through sensory stimulation-induced gamma oscillations. Singer et al. found that the application of 40-Hz visual-auditory combined stimulation was not only safe and well-tolerated in AD patients, but also preliminary results from magnetic resonance imaging and cerebrospinal fluid proteomics analysis affirmed the efficacy of this sensory stimulation in enhancing neural network connectivity and downregulating neuroinflammatory response factors [25]. Hajos et al. also revealed that 40-Hz visual-auditory combined stimulation was effective in enhancing sleep quality and daily life activities in AD patients [26]. However, there was a lack of clinical studies applying this method to post-stroke populations and understanding the underlying mechanism of the technique.

### Objectives {7}

This study aims to conduct a prospective clinical trial to validate the safety and effectiveness of the gamma oscillations induced by 40-Hz visual-auditory stimulation in treating patients with post-stroke upper limb dysfunction and explore the relevant mechanisms.

### Trial design {8}

**Fig. 1:**
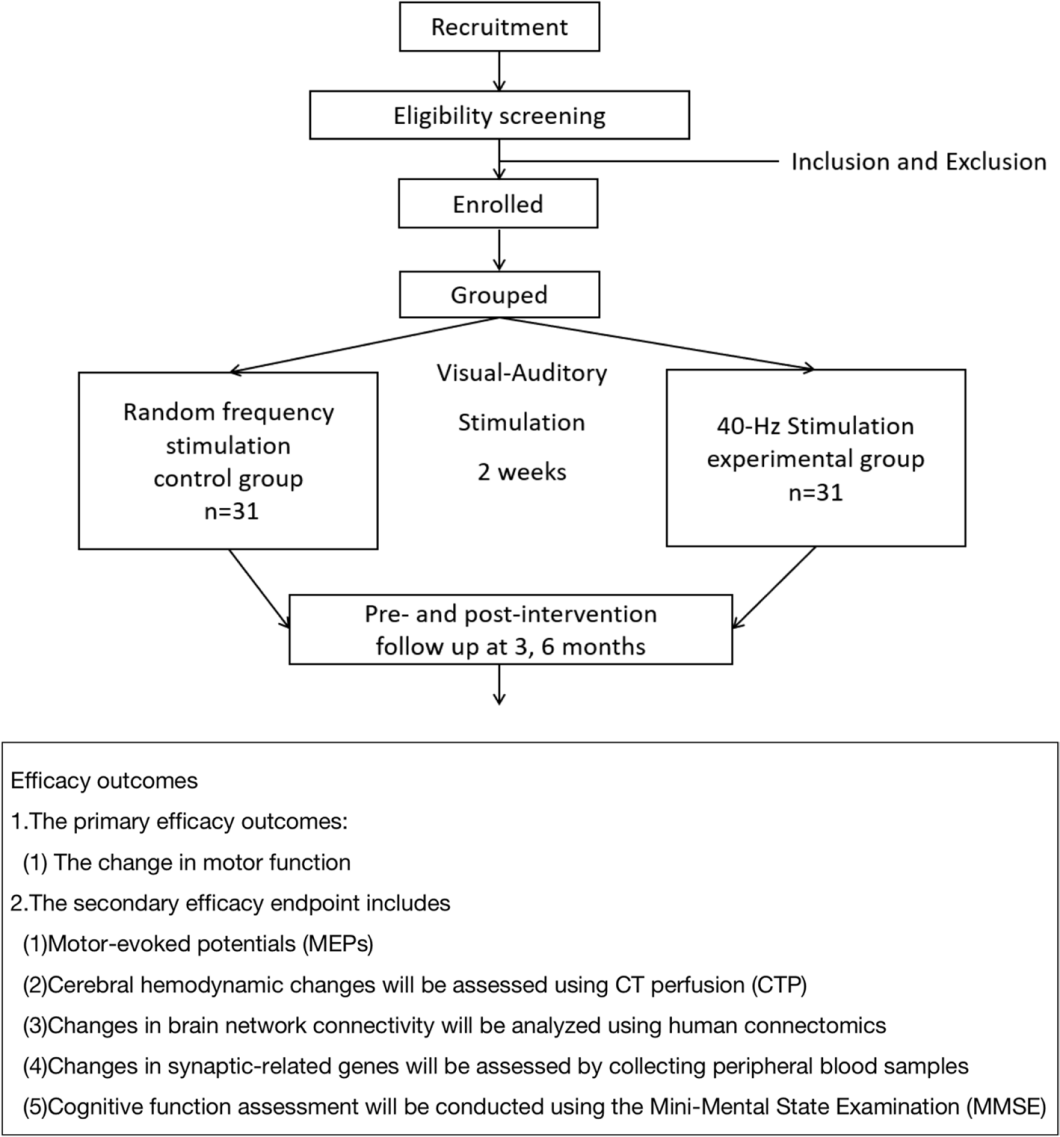
Comprehensive study flowchart of the protocol

This trial is a prospective, randomized controlled, double-blind superiority study with pre-intervention (− t1), 2 weeks post-intervention retention (T2), 3 months (T3), and post-intervention follow-up (T4) tests. The overall design is demonstrated in Fig. 1. Patients were randomly assigned to two parallel groups with a 1:1 ratio, with the experimental group receiving intervention through 40-Hz visual-auditory stimulation, and the control group undergoing intervention with randomly matched visual-auditory stimulation frequencies. A trained researcher, blinded to the group allocation, will collect the data which is shown in Table 1. The design of this trial is in accordance with the Standard Protocol Items.

**Table 1:**
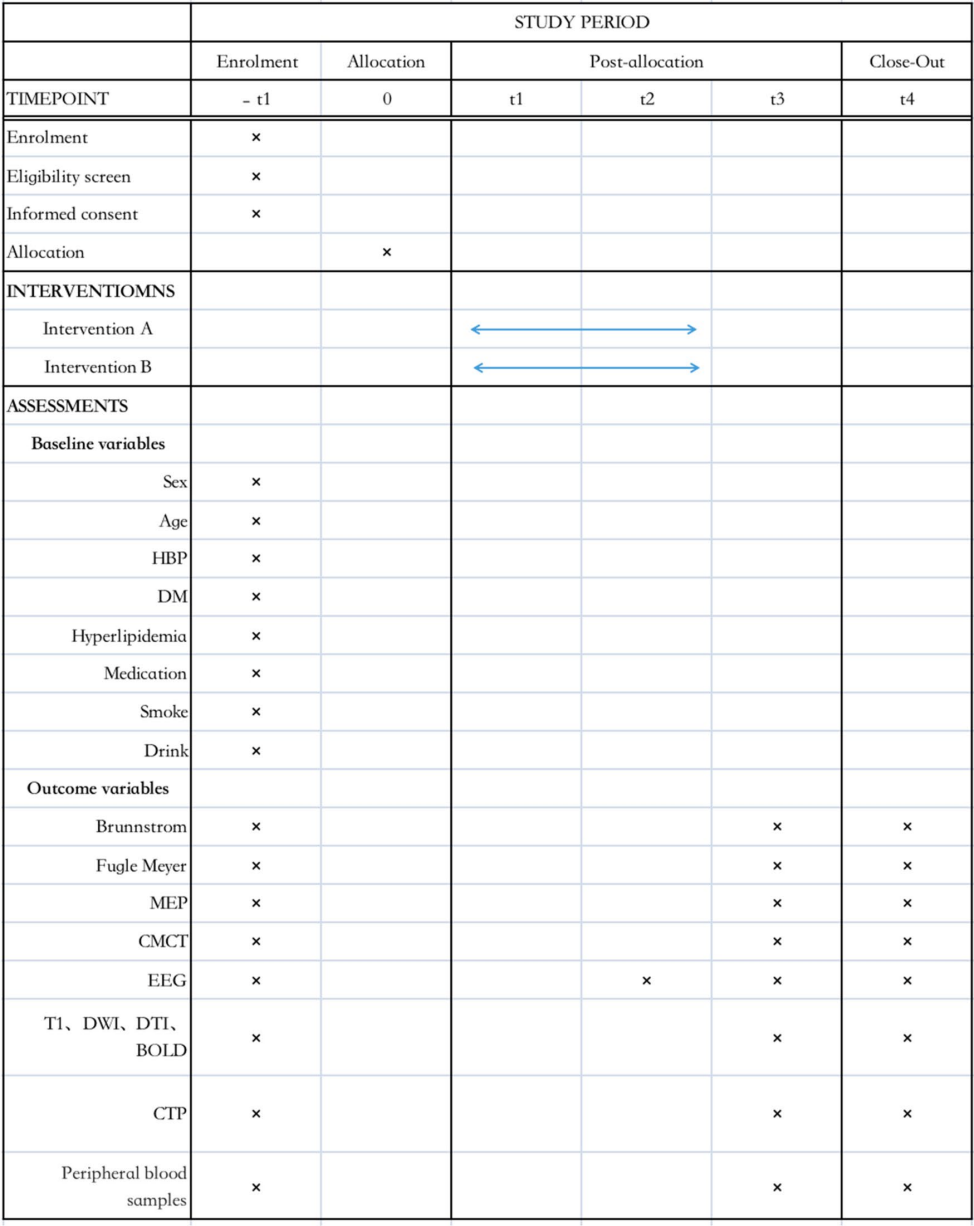
Schedule of enrollment and follow-up points

*Intervention A* 40-Hz visual-auditory stimulation group, *Intervention B* Random frequency visual-auditory stimulation group, *HBP* high blood pressure, *DM* diabetic mellitus, *MEP* motor-evoked potentials, *CMCT* central motor conduction time, *EEG* electroencephalography, *T1* T1 weighted, *DWI* T1 weighted, Diffusion-weighted imaging, *DTI* diffusion-weighted imaging, *BOLD* blood oxygen level dependent, *CTP* computer tomography perfusion, *T1* pre-intervention, *T2* 2 weeks post-intervention retention, *T3* 3 months, *T4* 6 months post-intervention follow-up

## Methods: participants, interventions, and outcomes

### Study setting {9}

A prospective inclusion of 62 stroke patients will be carried out from October 2023 to December 2024 at the Department of Neurology, Seventh People’s Hospital, affiliated with the Shanghai University of Traditional Chinese Medicine. Each patient will be enrolled into the study after signing an informed consent form.

### Eligibility criteria {10}

#### Inclusion criteria

1. Age ≥ 40 years.
2. Acute anterior circulation ischemic stroke patients meeting the following criteria:
  (2.1) Impaired contralateral limb motor function.
  (2.2) Paralysis of the affected limb with muscle strength rated at levels I–IV according to the Brunnstrom staging.
3. Onset of symptoms within 1 week.
4. Sign an informed consent form.

#### Exclusion criteria

1. Presence of acoustic-optical impairments that may hinder intervention and assessment.
2. Individuals with aphasia or severe cognitive impairments, making normal communication difficult (MMSE scores: < 17 for illiterate, < 20 for primary education, < 22 for secondary education, and < 23 for university education).
3. Inability to maintain an independent sitting position for at least 0.5 h or tolerate training and assessment for 1 h. Inability to tolerate visual-auditory stimulation.
4. Individuals who have used medications affecting cortical excitability (e.g., antiepileptic drugs, sedatives, or hypnotics) in the past 3 months.
5. Confirmed cases of mental illness, severe depression (with suicidal tendencies), or epilepsy, or a family history of mental illness or epilepsy.
6. Severe heart, liver, kidney diseases, or infectious diseases, or a life expectancy of less than 90 days.
7. Individuals with pre-existing motor impairments or abnormal muscle tone (MRS > 2 points).
8. Individuals with a history of prior traumatic or musculoskeletal disorders affecting functionality.
9. Individuals who have participated in or are currently involved in drug or device clinical trials within the last 30 days.
10. Pregnancy.

### Who will take informed consent? {26a}

The informed consent will be conducted by experienced neurologists and signed by two sides (blinded physicians and stroke patients). The potential participant has sufficient time to read the full text of the informed consent form, and neurologists will introduce the details of the contents of the informed consent form to them. The informed consent will be signed by the patient personally or the patient’s legal representative before the study.

### Additional consent provisions for collection and use of participant data and biological specimens {26b}

After the patient signs informed consent, blood will be sampled by venipuncture of elbow from participants. The entire blood sampling process is operated by a professional neurology nurse who extracts about 2 ml.

## Interventions

### Explanation for the choice of comparators {6b}

Current conventional treatment modalities for post-stroke upper limb dysfunction, including medication and routine rehabilitation, yield suboptimal results. Prior research has confirmed that 40-Hz visual-auditory stimulation can induce stable gamma oscillations. This study aims to explore the therapeutic effects of gamma oscillations on post-stroke upper limb dysfunction, building upon the aforementioned treatments. Therefore, the experimental group will receive 40-Hz visual-auditory stimulation to induce gamma oscillations, while the control group will receive randomly frequency-matched visual-auditory stimulation without inducing gamma oscillations. To monitor the induction of gamma oscillations during the intervention, this study will concurrently monitor electroencephalography (EEG) and analyze it to ensure that the experimental group exhibits induced gamma oscillations while the control group does not.

### Intervention description {11a}

All patients will receive treatment for 25 min per day, 7 days per week for 2 weeks (14 sessions). For all two interventions, the therapists will provide verbal instructions, cues, feedback, and help, when needed.

**Fig. 2:**
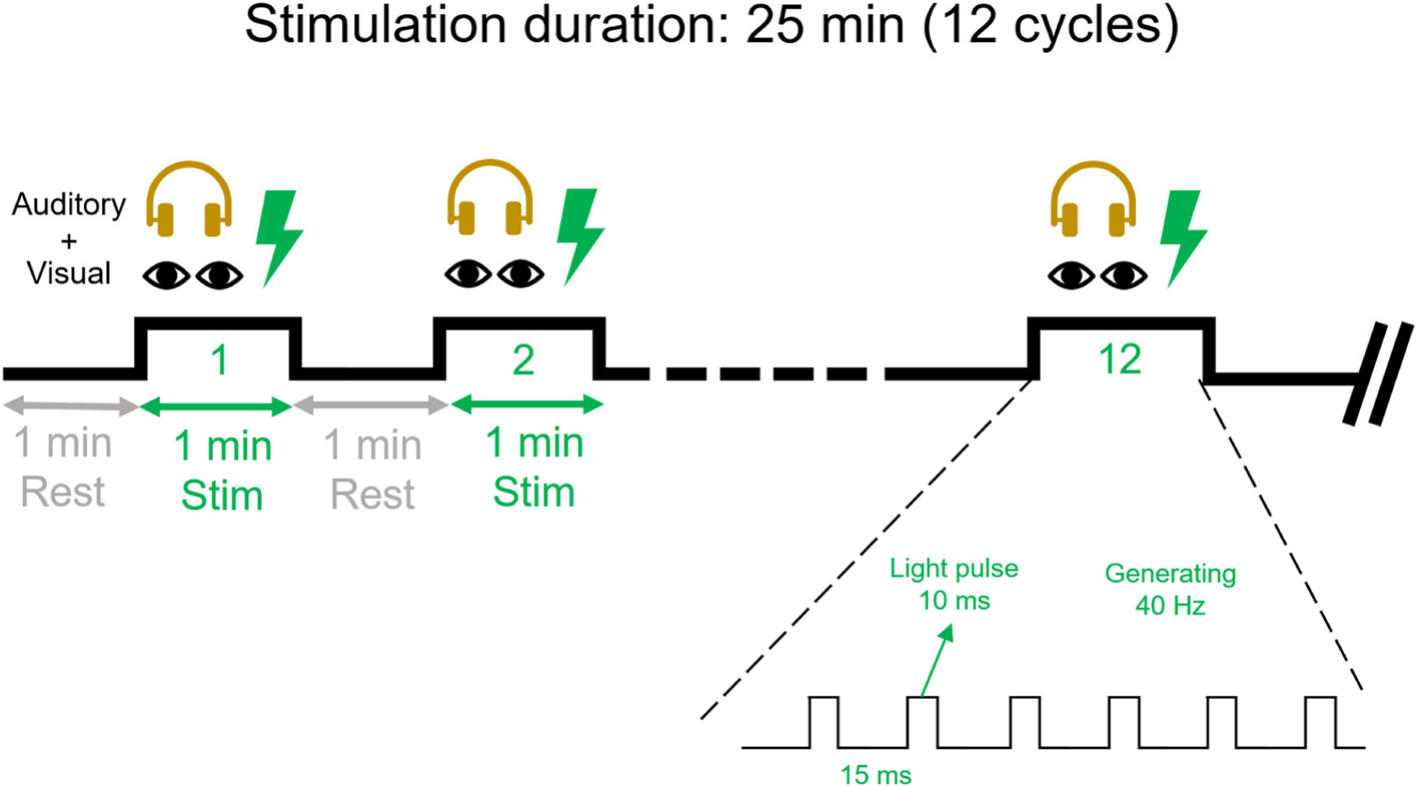
Detailed intervention protocol

At a distance of approximately 50 cm from the patient’s eyes, an LED light panel will be positioned. The patient will face to the LED light panel and wear Bluetooth headphones. In this study, an Arduino development board will be employed to control the LED light panel’s signal generator and the playback of specific frequency audio through the headphones. The intervention is demonstrated in Fig. 2

1. 40-Hz stimulation group

Each stimulus light pulse generated by the LED light panel will have a duration of 5 ms and a pulse repetition frequency of 40 Hz (40 pulses emitted per second with a pulse interval of 20 ms). The headphones will play 40-Hz audio, and each stimulation session will last for a duration of 1 min. The stimulation group will receive a total of 12 cycles of stimulation.

(2) Random frequency visual-auditory stimulation group

Each stimulus light pulse generated by the LED light panel will have a duration of 10 ms, and the pulse repetition frequency will vary randomly between 1 and 100 Hz (random emission of 1–100 pulses per second with random pulse intervals ranging from 5 to 995 ms). The headphones will play audio with random frequencies, and each stimulation session will last for a duration of 1 min. The group will receive a total of 12 cycles of stimulation.

Continuous electroencephalogram (EEG) monitoring will be conducted throughout the intervention period to record brain electrical activity.

### Criteria for discontinuing or modifying allocated interventions {11b}

There is no anticipated major problem that is detrimental to the participant. Participants can withdraw from the trial any time, and their routine treatment would not be affected. The allocated interventions can be modified when (1) participant requests or (2) the allocated intervention does no good to the participant’s disease. Immediately, communication with the relevant parties will be made by the trial principal investigators, as soon as any changes are made.

### Strategies to improve adherence to interventions {11c}

To ensure the protocol adherence of the intervention and assessment, all the neurologists, physiotherapists, nurses, and engineers involved in the research project will be trained according to the recommended guidelines and protocols.

### Relevant concomitant care permitted or prohibited during the trial {11d}

During the study, the stroke patients will be able to continue their daily treatment plan at the hospital as usual. However, other neuromodulation treatment methods, such as transcranial electrical stimulation, will be prohibited.

### Provisions for post-trial care {30}

There is no anticipated harm for visual-auditory stimulation intervention. However, if participants who feel uncomfortable, such as dizziness, nausea, and headache, will be immediately evaluated by the neurologist for further necessary treatment.

### Outcomes {12}

Pre-intervention (− t1), 2 weeks post-intervention retention (T2), 3 months (T3), and post-intervention 6 months follow-up (T4), cranial MRI scans (T1, T2, DWI, DTI, BOLD) and EEG will be employed to detect changes in neural network connectivity, Fugl-Meyer Assessment (FMA) and Brunnstrom Limb Function Recovery Scale will be used to assess motor function, transcranial magnetic stimulation (TMS) will be used to detect neurophysiological biomarkers including MEPs and CMCT, CT perfusion (CTP) will be employed to detect cerebral blood flow, and peripheral blood samples will be collected for RNA sequencing.

## Primary outcomes

### Fugl-Meyer Assessment (FMA)

Motor function recovery will be assessed using the Fugl-Meyer Assessment (FMA). The FMA rates each sub-item on a scale of 3, with scores of 0 (unable to complete), 1 (partially completed), and 2 (fully completed). The upper limb motor assessment comprises a total of 33 items, with a maximum score of 66.

### Brunnstrom Limb Function Recovery Scale

Motor function recovery will also be assessed using the Brunnstrom Limb Function Recovery Scale. The Brunnstrom Limb Function Recovery Scale classifies the upper limb and hand as Stage VI, with Stage I indicating no movement and Stage VI indicating normal or near-normal function.

## Secondary outcomes

### Motor-evoked potential (MEP)

The study will employ single-pulse transcranial magnetic stimulation (sTMS, Xiang Yu Medical; China) to the primary motor cortex (M1 area) of the hand region. This technique will measure motor-evoked potential (MEP) in the first dorsal interosseous muscle via surface electrodes. MEP amplitude, latency, resting motor threshold (RMT), active motor threshold (AMT), and central motor conduction time (CMCT) will be evaluated as indicators of corticospinal tract function. Subjects will be seated comfortably with their head and arm relaxed. The TMS coil will be placed 5 cm lateral to the vertex, aligning at a 45° angle from the brain midline with the handle pointing backwards. The location yielding consistently maximal MEP responses will be identified as the “motor hotspot” [27]. The magnetic stimulus intensity will be set at 20% above the MEP threshold. The TMS operator will adjust the magnetic cortical stimulus intensity to elicit MEPs of peak-to-peak amplitude 1 mV on average [28]. Five sequential stimulations will be administered at each intensity, and the average of the resulting five MEP traces will be used for data analysis. Latency will be reported as mean ± SD, while amplitude will be represented by the median due to data distribution skewness.

CMCT indicates the time it takes for a signal to travel from the motor cortex to spinal motor neurons. To measure CMCT, we will use spinal magnetic stimulation. After cortical stimulation, each participant will undergo cervical stimulation. The coil will be placed above the C7 spinous process, 2 cm lateral to the midline, aiming to activate cervical nerve roots at the intervertebral foramina. Participants will experience an MEP with a latency time from cortical magnetic stimulation of the first dorsal interosseous muscle. They will also experience an MEP with a different latency time due to cervical magnetic stimulation of the same muscle (peripheral motor conduction time). The difference between these two latency times provides the CMCT value.

### CT perfusion (CTP)

Cerebral hemodynamic changes will be assessed using CTP in cerebral blood flow before and after the intervention. The imaging examination utilized a Siemens SOMATOM Force 960 + model with a 320-row spiral scanner. Patients underwent CT plain scans initially, followed by cerebral perfusion imaging using dynamic perfusion mode. The scan parameters were set as follows: collimation width of 40 mm, tube voltage of 120 kV, tube current–time product of 320 m As, gantry rotation speed of 0.35 s per rotation, pitch of 1.2375, with 20 samples (each machine movement corresponds to two samples), a sampling interval of 1.5 s, and a scan length of 105 mm. After selecting the appropriate range, the MALLINCKRODT high-pressure injector was used for intravenous injection through the antecubital median vein. Lohexol injection (370 mg I/ml) at a rate of 5 ml/s, 50 ml in total, was administered, and the scan continued for 60 s, encompassing 20 cycles and yielding 640 slices. Upon completion of the scan, the images were automatically transferred to the United Imaging AI post-processing workstation (uai_pacs). All scan data were transferred to the uai_pacs post-processing workstation, and the Stroke Protocol within the CT Brain Perfusion Analysis software was employed for processing and analysis. The software automatically generated pseudo-color maps of cerebral perfusion based on the maximum lesion level for assessment. The regions of interest included the core infarction zone and the ischemic penumbra. The software automatically matched the mirrored zones on the healthy side, obtaining related data and pseudo-color maps, encompassing cerebral blood flow (CBF), cerebral blood volume (CBV), mean transit time (MTT), time to peak (TTP), ischemic penumbra (IP) volume, and other parameters related to perfusion, thereby providing perfusion color maps.

### Functional connectivity by magnetic resonance imaging (MRI)

We will use a 3-T MRI scanner (Siemens Skyra, Erlangen, Germany), along with a circular surface coil to investigate changes in the brain. This will include evaluations of gray matter density, cortical thickness, subcortical nuclei volumes, and functional connectivity. Each participant will undergo an MRI scan lasting approximately 25 min. During the scan, they will have their eyes closed and extra padding will be placed around their ears to reduce noise interference. We will analyze changes in neural network connectivity using human connectomics. By integrating intelligent imaging technology with multimodal imaging data, including T1, DTI, and BOLD functional MRI, we can compare alterations in brain fiber connectivity patterns and changes in brain functional regions before and after treatment. This approach allows us to visualize and quantify functional connections between brain regions, alterations in the number of nerve fiber bundles, changes in the direction of nerve fiber bundle pathways, and changes in the volume of functionally abnormal regions.

### Gamma oscillation entrainment via electroencephalogram (EEG)

In this study, a 32-channel scalp EEG signal recording system (Brain Amp MR PLUS, Brain Products, Germany) with a sampling rate of 500 Hz will be utilized. The electrode positions will be based on the international 10/20 system, with CPz as the reference electrode and AFz as the ground electrode. Prior to the experiments, the impedance of all electrode channels will be adjusted to below 20 kΩ. Preprocessing of data from all subjects will be done using the Makoto pipeline and EEG Lab toolbox in MATLAB. We will assess gamma oscillation entrainment by calculating changes in power spectral density before, during, and after the intervention using custom MATLAB functions.

### RNA sequencing of the blood sample

Every participant will have a 1-mL blood sample taken from a vein by a trained nurse both at the start of the study and 2 weeks after the intervention period. This blood collection will allow us to assess changes in neuroinflammatory factors, nerve growth factors, and genes related to synaptic plasticity in the blood. Prior to the blood draw, participants will be advised to refrain from eating after 8 pm the previous day, as well as drinking and engaging in intense exercise. Breakfast will also be prohibited on the day of the blood draw. Once they have emptied their bladder, they will proceed to a laboratory room where a 1-mL venous blood sample will be collected into an EDTA anticoagulant tube. To this, 3 mL of TRIzol Reagent (LMAl Bio, China) will be added, ensuring thorough mixing. The mixture will then be incubated at 25 °C for 5 min and stored at − 80 °C. This rigorous process maintains the integrity and stability of the blood samples for subsequent biochemical analysis.

### Participant timeline {13}

Time schedule of enrolment, interventions, assessments, and visits for participants is shown in Table 1.

### Sample size {14}

The sample size calculation was conducted using G*power software (v3.1.9.2). The effect size of this study was estimated from a study conducted by Kaux et al. [29] who investigated the effects of transcranial direct current stimulation associated with physical therapy in acute stroke patients evaluated by the upper extremity section of the Fugl-Meyer Test, which was determined to be 0.995. The experiment employs a 1:1 simple randomization, n1 = n2. According to a prior difference between two independent means analysis *t* test, with a power of 0.95 and an error probability of 0.05, we applied these values to compute a sample size of *n* = 28. Accounting for a 10% attrition rate, we determined that each group should comprise 31 participants, yielding a total of 62 stroke patients included in the study. It is important to note that all enrolled patients received clinical medications and conventional rehabilitation treatments that were generally uniform.

### Recruitment {15}

The project is based at the Department of Neurology and Neurorehabilitation of the Seventh People’s Hospital affiliated with Shanghai University of Traditional Chinese Medicine. This hospital is a national comprehensive stroke and a member of the national neurology clinical research centre. It possesses outstanding capabilities in acute stroke management and post-stroke functional rehabilitation. Over the past 3 years, it has treated more than 1000 cases of stroke, guaranteeing the enrolment of participants.

## Assignment of interventions: allocation

### Sequence generation {16a}

Participants will be allocated by simple randomization. The random number will be generated by SPSS version 20.0 for Windows (IBM Co., Armonk, NY, USA). The participants will be sample randomized into one of the two treatment groups (40-Hz stimulation vs. random frequency stimulation) in a 1:1 ratio after baseline evaluation.

### Concealment mechanism {16b}

The randomization code generated by the computer will be sealed in an opaque, sealed envelope and is only available to the physician who performs the intervention and not involved in the outcome evaluations or patient follow-ups. Member responsible for patient enrollment, outcome evaluation, and follow-up are blind to the allocation sequence.

### Implementation {16c}

The primary investigators will be responsible for enrollment of participants. The enrolled patients will be assigned to interventions according to the computer-generated random numbers.

## Assignment of interventions: blinding

### Who will be blinded {17a}

Procedures will be implemented to control for expectancy effects related to visual-auditory stimulation. Persons who will be masked to visual-auditory stimulation status (40-Hz stimulation vs. random frequency stimulation) are participants, authorized representative of the patient (if applicable), personnel involved in outcome assessments, follow-up, data collection, and analysis. Unmasked personnel will be the engineers who set up the stimulation protocol for the device and physicians who perform the intervention.

### Procedure for unblinding if needed {17b}

We do not anticipate any requirement for unblinding unless it is relevant to the participant safety.

## Data collection and management

### Plans for assessment and collection of outcomes {18a}

The physician who performed the intervention is qualified and adequately trained about visual-auditory stimulation, and he has performed the intervention more than 50 patients. All outcomes will be evaluated by two experienced neurologists who are blind to the grouping of the patients. All imaging examinations will be completed in the same machine.

### Plans to promote participant retention and complete follow-up {18b}

We will make every effort to lower the obstacles to attending study visits, including offering the participants to come at preferred times and offering remote options for non-face-to-face study tasks via videochat.

### Data management {19}

Each patient’s ID and baseline information will be collected. Collected data will be recorded on paper case report forms (CRFs), then entered into electronic data capture (EDC) system and uploaded to a central server. Data integrity will be enforced through appropriate range checks and consistency checks at the time of data entry, before the data are committed to the database. Data entered into the database will be retrievable for viewing through the data entry application. A complete back up of the primary database will be performed once a week and stored indefinitely on a twin server. The CRFs and EDC will be kept for at least 5 years after publication in case of any inquiry. Personal information about the enrolled participants will be safely and confidentially kept. After completion of the study, the CRFs and all the data collected will be stored anonymously in the password-protected central server and restricted to relevant members of the research team. Paper copies of the CRFs will be stored in a locked cabinet in the relevant research office.

### Confidentiality {27}

Personal information about the enrolled participants will be safely and confidentially kept. After completion of the study, the CRFs and all the data collected will be stored anonymously in the password-protected central server and restricted to relevant members of the research team. Paper copies of the CRFs will be stored in a locked cabinet in the relevant research office.

### Plans for collection, laboratory evaluation and storage of biological specimens for genetic or molecular analysis in this trial/future use {33}

The blood sample of the participants will be collected by professional nurses. The samples will be mixed with Trizol solution at a ratio of 1:3 and stored in a − 80 ℃ freezer at the central laboratory of Shanghai Seventh People’s Hospital before sending to RNA sequencing.

## Statistical methods

### Statistical methods for primary and secondary outcomes {20a}

Statistical analysis was conducted using GraphPad Prism version 8.0 and MATLAB 2021a. For continuous variables, the normality of the data distribution in all datasets was assessed using the Kolmogorov–Smirnov test. Continuous variables were presented as mean ± standard deviation (SD) with range values for normally distributed data, or as the medians with the interquartile ranges (IQRs) for non-normally distributed data, respectively. Categorical variables were presented as frequencies and percentages. If the data followed a normal distribution, the difference between the two groups was assessed using Student’s *t* test. If the data did not follow a normal distribution, the Wilcoxon rank test was employed. For categorical variables, a chi-squared test or Fisher’s exact test was utilized for comparison. A significance level of *P* < 0.05 was considered statistically significant.

### Interim analyses {21b}

No interim analyses are prepared for this trial as there are no anticipated problems that are detrimental to the participants.

### Methods for additional analyses (e.g., subgroup analyses) {20b}

There are no additional analyses except for interim analysis for the trial.

### Methods in analysis to handle protocol non-adherence and any statistical methods to handle missing data {20c}

As we mentioned above that we will keep in contact with all participants at least once a week, then we will know the conditions of the participants. Meanwhile, all the participants will be followed at scheduled time to ensure the adherence. The missing data will be handled by a single imputation model for analyses.

### Plans to give access to the full protocol, participant-level data, and statistical code {31c}

Data can be provided to researchers upon request, subject to a review of privacy. Requests for data can be sent to the corresponding author by email. Also all the data will be uploaded in the website of Chinese Clinical Trial Registry.

## Oversight and monitoring

### Composition of the coordinating center and trial steering committee {5d}

The principal investigators (PIs) of this clinical trial are PI # 1, Cong Wang, PhD, at the Shanghai Seventh People’s Hospital, Shanghai University of Traditional Chinese Medicine, and PI # 2, Feng Wang, PhD, at the Shanghai Seventh People’s Hospital, Shanghai University of Traditional Chinese Medicine. PI #1 is responsible for designing the research project’s concept, including the stimulation protocol, synaptic-related genes, EEG, and all neurophysiological recording methods. PI #1 will also perform and analyze all neurophysiological recordings for all study participants of the clinical trial along with her research team. PI #2 is responsible for designing radiological and clinical assessments, leading the implementation of visual-auditory stimulation and regular rehabilitation training, as well as conducting clinical assessments for study participants at the hospital. The trial steering committee comprises the PIs and dedicated research staff.

The PIs and dedicated research staff will form the trial steering committee, which is responsible for overseeing and managing the entire project. The study governance for this single-site study is divided into several teams, including the oversight team, recruitment team, intervention deployment and assessment team, neurophysiological recording and neuroimaging team, blood sample team, and data management and analysis team. The oversight team, led by the PIs, has overall responsibility for the conduct and progress of the study. Each team is led by a dedicated research staff or clinician and works closely with the oversight team to establish and monitor standard operating procedures. Each team meets weekly with the PI to discuss decisions and progress within their specific area of responsibility. Full study meetings are held quarterly and as needed to ensure all aspects of the study are coordinated and progressing according to plan.

### Composition of the data monitoring committee, its role and reporting structure {21a}

As visual-auditory stimulation technique is a low-risk intervention which has no substantial safety issues, there will not be a data monitoring committee.

### Adverse event reporting and harms {22}

Due to the low-risk nature of the trial, no adverse event (AE) is anticipated. For very rare cases such as seizure, nausea between study enrollment and hospital discharge will be reported to the Shanghai Seventh People’s Hospital Review Board. The visual-auditory stimulation intervention will be discontinued promptly and professional clinician will provide treatment.

### Frequency and plans for auditing trial conduct {23}

The Ethic Committee will perform ongoing regulatory monitoring of the study, including planned site visits and trial dataset exploration. The trial steering committee and Ethics Committee will meet annually to review the trial’s conduct and progress, beginning in the study’s initial year.

### Plans for communicating important protocol amendments to relevant parties (e.g., trial participants, ethical committees) {25}

Immediately, communication with the ethical committee and the participants will be made by Dr Feng Wang, trial principal investigator, as soon as any changes are made.

### Dissemination plans {31a}

Final findings will be disseminated through presentations at scientific conferences or publications in peer-reviewed journals.

## Discussion

The treatment methods of stroke have evolved. However, the challenge of reducing disability and enhancing the quality of life for stroke patients while saving lives remains a pressing concern. Effective rehabilitation therapy plays a critical role in restoring health and reducing the burden of the disease. Moreover, investing in stroke rehabilitation yields a favorable cost-effectiveness advantage [30]. In daily life, complex motor activities are primarily executed by the upper limbs, and its impairment is a significant barrier for patients to reintegrate into normal social activities. Although there are numerous rehabilitation methods, they have yet to significantly ameliorate upper limb functional impairments. Therefore, new rehabilitation approaches are needed.

Neuronal oscillations refer to rhythmic electrophysiological activities in the brain that are considered highly correlated with information processing and represent a characteristic feature of brain states. Among these, gamma neuronal oscillations within the frequency range of 30–100 Hz play a crucial role in processes such as neural plasticity, neural circuits, and information transmission [7]. Stimulating gamma oscillations within the gamma frequency range has shown to promote brain function recovery. Various methods are employed for inducing gamma oscillations, with commonly used techniques including transcranial electrical stimulation and repetitive transcranial magnetic stimulation. However, these methods are limited by the lack of clarity in target area selection, the unclear dose–response relationship in neural circuit activation and reconstruction, and the instability of gamma oscillations induced in the brain due to the non-sinusoidal nature of rTMS pulse sequences and the associated high sound levels [19]. Visual-auditory stimulation offers a non-invasive, effective, and widely applicable method for inducing stable gamma oscillations. This approach is characterized by its simplicity, convenience, and repeatability. Previous research has reported that using 40-Hz visual-auditory stimulation can induce gamma neural oscillations, which, in turn, promote neural plasticity and enhance cognitive function recovery in AD mouse models [21, 22, 25, 31]. The clinical trials conducted by Tsai’s research group in AD have demonstrated the safety, tolerability, and feasibility of long-term gamma frequency visual-auditory stimulation. These initial findings suggested that this modulatory approach exhibits immunological and network effects [23]. However, its effectiveness in the context of upper limb functional recovery post-stroke remains unclear.

Preliminary experiments have demonstrated that 40-Hz visual-auditory stimulation can effectively improve motor function in stroke-afflicted mice. The underlying mechanism involves the restructuring of the brain’s neural network. The brain’s neural network comprises functional networks, reflecting the brain’s activity during rest or various states, and structural networks, represented by neural fibers within the brain. In 2016, the Human Connectome Project (HCP), funded by the National Institutes of Health (NIH) in the USA, established the Human Connectome Project Multimodal Parcellation (HCP-MMP1) [32]. This achievement produced a neural map that integrates both brain anatomical structure and brain function.

In previous research, our team utilized advanced neuroimaging techniques and, following the HCP parcellation method, integrated structural and functional neural networks to create multimodal fusion images that depict brain functionality. This approach combines structural, functional, and connectivity patterns, allowing for a more precise and personalized analysis of changes in brain neural networks. It is important to note that endogenous neural repair mechanisms are initiated within hours after a stroke occurs, reaching their peak at around 10 weeks and then gradually slowing down, eventually entering a plateau phase after 3 months [33]. This underscores the critical importance of the acute-phase post-stroke period for neural function protection and remodeling.

Based on this, our project aims to prospectively include patients with post-stroke upper limb motor impairment. We will employ 40-Hz visual-auditory stimulation to induce gamma oscillations and explore its effects on improving limb function. Simultaneously, we will investigate evidence of enhanced neuroplasticity within the brain’s neural circuits, connectivity, and cerebral blood flow. This approach will help us unravel the relationship between gamma oscillations and the remodeling of the brain’s neural networks. This non-invasive neuromodulation method, the 40-Hz visual-auditory stimulation, will provide a theoretical foundation for its application in acute-phase post-stroke upper limb rehabilitation. Furthermore, it will pave the way for innovative approaches in the large-scale clinical application of this neuromodulation method in the field of stroke recovery.

## Trial status

This trial has been registered in the Chinese Clinical Trial (ChiCTR2300076579) on October 12, 2023, after approval of the Ethics Community of Shanghai Seventh People’s Hospital, Shanghai University of Traditional Chinese Medicine (2023-7th-HIRB-027) on May 8th, 2023. The inclusion began on October 15, 2023, and 2 patients in the treatment group have been included. Inclusion will be continued until the end of 2024, and the follow-up will be finished till 2024. The current trial protocol version is 1.0, and the informed consent version is 2.0.

## Data Availability

The principal investigators, Feng Wang and Cong Wang, will have access to the final trial dataset. Data can be provided to researchers upon request, subject to a review of privacy. Requests for data can be sent to the corresponding author by email.

## Abbreviations

rTMS: Repetitive transcranial magnetic stimulation
TMS: Transcranial magnetic stimulation
UE: Upper extremity
AD: Alzheimer’s disease
MMSE: Mini-Mental State Examination
MRS: Modified ranking scale
EEG: Electroencephalography
FMA: Fugl-Meyer Assessment
MEPs: Motor-evoked potentials
CTP: CT perfusion
RMT: Resting motor threshold
AMT: Active motor threshold
CMCT: Central motor conduction time
MAE: Major adverse events
DHI: Dizziness Handicap Inventory
AE: Adverse event
ADE: Adverse device effect
CRF: Case report form
EDC: Electronic data capture
